# Seroprevalence of arthropod-borne bacterial infections in homeless individuals in Hamburg in 2020

**DOI:** 10.1007/s15010-023-02059-y

**Published:** 2023-06-08

**Authors:** Fabian Heinrich, Jessica Rauch, Franziska Bertram, Volkhard A. J. Kempf, Silke Besier, Piotr Kuta, Thomas Renné, Benjamin Ondruschka, Klaus Püschel, Dennis Tappe

**Affiliations:** 1 Institute of Legal Medicine, University Medical Center Hamburg-Eppendorf, Hamburg, Germany; 2 Bernhard Nocht Institute for Tropical Medicine and National Reference Center for Tropical Pathogens, Hamburg, Germany; 3 Institute for Medical Microbiology and Infection Control and German Consiliary Laboratory for Bartonella, Frankfurt, Germany; 4 Institute of Clinical Chemistry and Laboratory Medicine, University Medical Center Hamburg-Eppendorf, Hamburg, Germany; 5 Irish Centre for Vascular Biology, School of Pharmacy and Biomolecular Sciences, Royal College of Surgeons in Ireland, Dublin, Ireland; 6 Center for Thrombosis and Hemostasis (CTH), Johannes Gutenberg University Medical Center, Mainz, Germany

**Keywords:** Rickettsia, Coxiella, Francisella, Bartonella, Ectoparasites

## Abstract

**Purpose:**

The number of homeless people in Germany is steadily increasing. Due to their often precarious living conditions, this specific population may be increasingly exposed to ectoparasites that can transmit various pathogens. To assess the prevalence and thus the risk of such infections, we analyzed the seropositivity of rickettsiosis, Q fever, tularemia and bartonellosis in homeless individuals.

**Methods:**

A total of 147 homeless adults from nine shelters in Hamburg, Germany, were included. The individuals underwent questionnaire-based interviewing, physical examination, and venous blood was drawn between May and June 2020. Blood samples were analyzed for antibodies against rickettsiae (*Rickettsia typhi* and *R. conorii*), *Coxiella burnetii*, *Francisella tularensis* and bartonellae.

**Results and conclusion:**

A very low seroprevalence of *R. typhi* and *F. tularensis* infection was found (0–1%), while antibodies against *R. conorii* and *C. burnetii* were more common (7% each), followed by a relatively high seroprevalence of 14% for bartonellosis. Q fever seroprevalence was associated with the country of origin, whereas bartonellosis seroprevalence was associated with the duration of homelessness. Preventive measures targeting ectoparasites, especially body lice, should be put in place continuously.

**Supplementary Information:**

The online version contains supplementary material available at 10.1007/s15010-023-02059-y.

## Introduction

In Germany, estimated 417.000 people suffer from homelessness [1], with numbers continuously rising. Not only is this group exceptionally vulnerable, but homeless people are more intensely exposed to pathogens and spread infections within their environment more easily [2]. Crowded living and poor hygienic conditions might lead to increased contact of homeless people with ectoparasites, such as lice, fleas and ticks, as well as urban rodents [3], paving the way for vector-transmitted infectious diseases [4]. While varying prevalences of arthropod-borne bacterial infections have been reported in homeless individuals mostly from the United States and southern Europe [3, 5], systematic seroprevalence analyses for these diseases in homeless are scarce in more temperate regions of Europe. Here, we report on the seroprevalences of different arthropod-borne bacterial pathogens in 147 homeless individuals in the metropolitan area of Hamburg, Germany.

## Patients and methods

A total of 147 homeless adults from nine shelters in Hamburg, Germany, were included. Shelters housed 5–35 (median 14) participants each. The individuals underwent questionnaire-based interviewing, physical examination, and venous blood was drawn between May and June 2020. The study was approved by the ethics committee of the Hamburg Medical Board (no. PV7333). Items of the questionnaire comprised age, sex, country of origin, time of homelessness, and sleeping outside homeless assistance facilities. Body temperature was measured using a certified tympanic thermometer (Genius 3, CardinalHealth, Ohio).

Blood samples were analyzed for white blood count as marker for acute infection (Advia 2120i, Siemens Healthcare, Erlangen, Germany). Antibodies against rickettsiae (*Rickettsia typhi* and *R. conorii*) were measured by in-house indirect immunofluorescence tests (IFAT [6]), and antibodies against *Coxiella burnetii* by a commercial IFAT (Vircell, Granada, Spain). Seroprevalence of *Francisella tularensis* infection was analyzed by enzyme immunoassay (EIA) and immunoblot (Seramun Diagnostica, Heidesee, Germany), and of bartonellosis by EIA (NovaTec, Dietzenbach Germany [7]) and confirmed by IFAT (Euroimmun, Lübeck, Germany).

For serological test validation and verification purposes of the respective infections, 30–50 blood samples of healthy blood donors were analyzed, and background seroprevelance data were obtained.

Statistical analyses were performed with STATA/MP 17.0 (StataCorp, Texas). For continuous variables, median and interquartile range (IQR), and for categorical variables, percentages and numbers are illustrated. Continuous variables were compared using the Mann–Whitney–U test, and categorical variables were compared using Chi-square and Fisher's exact test, as appropriate.

## Results

Of the 147 homeless individuals analyzed, 20% were female (n = 29). The median age was 46 years (IQR: 35–55), and the predominant proportion of homeless individuals was born in Germany (n = 61, 44%). Of the individuals included, 3/147 (2%) suffered from sub-febrile to febrile temperatures (tympanic temperature > 37.5 °C), and 7/147 (5%) had laboratory-confirmed leukocytosis (> 10.000 WBC/µL).

A seroprevalence of infections with *R. conorii* (7% [95% CI: 3–12], n = 10/147), *R. typhi* (0% [95% CI: 0–2]; n = 0/147), *C. burnetii* (7% [95% CI: 3–12]; n = 10/147), *F. tularensis* (1% [95% CI: 0–4], n = 1/147), and *Bartonella* spp. (14% [95% CI: 9–21], n = 21/147) was found (Table 1; Supplementary Tables). Seroprevalence in healthy blood donors from Hamburg for the respective infections was below 1% each, except for bartonellosis (3.5%). Of the three individuals with fever, one had both antibodies against *R. conorii* (IgM and IgG) and *C. burnetii* (phase I and II IgM and IgG), the second exhibited antibodies against bartonellae (IgG), and the third showed none of the antibodies tested.

**Table 1:** Baseline characteristics of homeless individuals included in the study with individuals grouped according to their serostatus for common arthropod-borne bacterial infections. Valid percentages are illustrated

	Total homeless individuals	*R. conorii* Seropositive	*C. burnetii* seropositive	*F. tularensis* seropositive	*Bartonella* sp. seropositive
		N = 10	N = 10	N = 1	N = 21
Median age [years] (IQR)	46.0 (35.0–55.0)	51.0 (47.0–55.0)	54.5 (46.0–60.0)	37.0 (37.0–37.0)	46.0 (40.0–54.0)
Sex					
Male	117 (80.1%)	7 (70.0%)	9 (90.0%)	1 (100.0%)	19 (90.5%)
Female	29 (19.9%)	3 (30.0%)	1 (10.0%)	0 (0.0%)	2 (9.5%)
Country of origin					
Germany	61 (44.2%)	4 (40.0%)	1 (10.0%)	0 (0.0%)	8 (40.0%)
Directly neighboring Countries	41 (29.7%)	4 (40.0%)	3 (30.0%)	1 (100.0%)	10 (50.0%)
Non-directly neighboring Countries	36 (26.1%)	2 (20.0%)	6 (60.0%)	0 (0.0%)	2 (10.0%)
Time of homelessness [months] (IQR)	18.0 (5.0–60.0)	12.0 (3.0–144.0)	36.0 (7.0–480.0)	84.0 (84.0–84.0)	54.0 (8.0–132.0)
Sleeping in homeless assistance facilities	103 (75.7%)	9 (90.0%)	7 (77.8%)	1 (100.0%)	15 (78.9%)

Seropositivity for two pathogens was found in 4/147 individuals (3%), of which a single participant was seropositive for *R. conorii* and *C. burnetii* and three for *Bartonella* spp. and *C. burnetii.* Seropositivity for *C. burnetii* was associated with the country of origin and *Bartonella* spp. with the time of homelessness; no other statistically significant association was found.

## Discussion

In this report, we analyzed seroprevalences of infections with several ectoparasite-transmissible bacterial pathogens in homeless individuals in a temperate metropolitan region. Not all of the pathogens are strictly associated with arthropods. While *C. burnetii* and *F. tularensis* may be transmitted by ticks, infection by inhalation and through skin injuries after exposure to dead hares, respectively, is usually more common. *B. quintana* is transmitted by body lice, while *B. henselae* is associated with cat scratches. However, the pathogen may also be transmitted by cat fleas. As homeless people are not always staying in shelters, but may dwell in urban parks and other grass and shrub surroundings, the seroprevalence of a spotted fever/tick-bite rickettsiosis was also investigated.

Antibodies against *R. typhi* were absent in homeless individuals analyzed in Hamburg, consistent with previously reported low seroprevalences in a French cohort living in a metropolitan region [5]. However, in outbreak situations, prevalences can reach 21.7% [5]. The seroprevalence of tick-borne *R. conorii* infections of 7% measured in this study exceeds previously reported ranges of 0.4–4.5% [5, 8]. Seropositivity for *R. conorii* was neither associated with sociodemographic characteristics, country of origin, time of homelessness, nor sleeping outside homeless assistance facilities. While sleeping outside was expected to expose homeless individuals to various ectoparasites, no association was found here. However, as serological cross-reactions between the spotted fever/tick-bite rickettsioses are immense, this seroprevalence here likely also encompasses rickettsioses found in more northern, temperate regions of Europe, such as *R. helvetica* infections and others. Likewise to tick-bite rickettsioses, the seroprevalence of *C. burnetii* infection was relatively high (7%) compared to previous publications among homeless (2.1%; [5]). Seropositivity in our study was associated with originating from countries not neighbouring Germany directly, primarily constituting people from eastern Europe (50%, n = 3/6). Interestingly, compared to other European countries, no increased infection rates were reported in eastern Europe itself in 2019 [9]. A seroprevalence of 1% for *F. tularensis* infection, comparable to previous studies among homeless [5], was found in our investigation. While camping has already been shown to be a risk factor for tularemia, no risk association was found with sleeping outside homeless assistance facilities. In accordance with prior studies in homeless [3, 5], seroprevalence for bartonellosis was 14% [3, 5] and might be due to contact with body lice. However, we did not serologically discriminate between infections with various *Bartonella* species. Seropositivity was associated with a longer period of homelessness (12 vs 54 months, p = 0.02) in our investigation.

As this was a point prevalence study, it remains challenging to determine whether individuals in this cohort had acute infections with the respective pathogens. However, in one patient with fever, IgM and IgG against both *R. conorii* and *C. burnetii* was found, likely reflecting acute disease.

In conclusion, we detected high seroprevalence for bartonellosis in homeless people in Hamburg, which was associated with the time of homelessness. Preventive measures, such as the provision of clean sleeping conditions and ensuring that fabrics are free of body lice, should be put in place continuously. While the seropositivity for *C. burnetii* was associated with the country of origin, no other statistically significant associations were found. Future studies with larger cohorts and in different metropolitan areas will expand these findings and take contacts to ectoparasites and rodents into account.

### Supplementary Information

Below is the link to the electronic supplementary material.

Supplementary file1 (DOCX 46 KB)

## Data Availability

The datasets generated and analysed during the current study are available from the corresponding author on reasonable request.
