# Supplementary material for: Seroprevalence of arthropod-borne bacterial infections in homeless individuals in Hamburg in 2020
Source: Infection. 2023 Jun 8;51(6):1819–22. doi: 10.1007/s15010-023-02059-y (PMC10665224; doi:10.1007/s15010-023-02059-y)
Supplement: Supplementary file 1 — Supplementary file1 (DOCX 46 KB) [file 15010_2023_2059_MOESM1_ESM.docx]

**Supplementary Table 1.** Baseline characteristics of homeless individuals included in the study, with individuals grouped by serostatus to *R. conorii*. Valid percentages are illustrated.

|  | ***R. conorii* seronegative** | ***R. conorii***  **seropositive** | **Comparative statistics**  **(p values)** |
| --- | --- | --- | --- |
|  | N=137 | N=10 |  |
| **Median age (IQR) [years]** | 45.0 (34.5-54.5) | 51.0 (47.0-55.0) | 0.11 |
| **Sex** |  |  | 0.42 |
| **Male** | 110 (80.9%) | 7 (70.0%) | . |
| **Female** | 26 (19.1%) | 3 (30.0%) | . |
| **Country of origin** |  |  | 0.77 |
| **Germany** | 57 (44.5%) | 4 (40.0%) | . |
| **Directly neighboring countries** | 37 (28.9%) | 4 (40.0%) | . |
| **Non-directly neighboring countries** | 34 (26.6%) | 2 (20.0%) | . |
| **Time of homelessness [months]** | 21.0 (5.0-60.0) | 12.0 (3.0-144.0) | 0.86 |
| **Sleeping in homeless assistance facilities** | 94 (74.6%) | 9 (90.0%) | 0.45 |

**Supplementary Table 2.** Baseline characteristics of homeless individuals included in the study, with individuals grouped by serostatus to *C. burnetii*. Valid percentages are illustrated.

|  | ***C. burnetii* seronegative** | ***C. burnetii* seropositive** | **Comparative statistics**  **(p values)** |
| --- | --- | --- | --- |
|  | N=137 | N=10 |  |
| **Median age (IQR) [years]** | 45.0 (35.0-54.0) | 54.5 (46.0-60.0) | 0.10 |
| **Sex** |  |  | 0.69 |
| **Male** | 108 (79.4%) | 9 (90.0%) | . |
| **Female** | 28 (20.6%) | 1 (10.0%) | . |
| **Country of origin** |  |  | 0.02 |
| **Germany** | 60 (46.9%) | 1 (10.0%) | . |
| **Directly neighboring countries** | 38 (29.7%) | 3 (30.0%) | . |
| **Non-directly neighboring countries** | 30 (23.4%) | 6 (60.0%) | . |
| **Time of homelessness [months]** | 15.0 (5.0-60.0) | 36.0 (7.0-480.0) | 0.23 |
| **Sleeping in homeless assistance facilities** | 96 (75.6%) | 7 (77.8%) | 1.00 |

**Supplementary Table 3.** Baseline characteristics of homeless individuals included in the study, with individuals grouped by serostatus to *F. tularensis*. Valid percentages are illustrated.

|  | ***F. tularensis* seronegative** | ***F. tularensis* seropositive** | **Comparative statistics**  **(p values)** |
| --- | --- | --- | --- |
|  | N=146 | N=1 |  |
| **Median age (IQR) [years]** | 46.0 (35.0-55.0) | 37.0 (37.0-37.0) | 0.49 |
| **Sex** |  |  | 1.00 |
| **Male** | 116 (80.0%) | 1 (100.0%) | . |
| **Female** | 29 (20.0%) | 0 (0.0%) | . |
| **Country of origin** |  |  | 0.56 |
| **Germany** | 61 (44.5%) | 0 (0.0%) | . |
| **Directly neighboring countries** | 40 (29.2%) | 1 (100.0%) | . |
| **Non-directly neighboring countries** | 36 (26.3%) | 0 (0.0%) | . |
| **Time of homelessness [months]** | 15.0 (5.0-60.0) | 84.0 (84.0-84.0) | 0.30 |
| **Sleeping in homeless assistance facilities** | 102 (75.6%) | 1 (100.0%) | 1.00 |

**Supplementary Table 4.** Baseline characteristics of homeless individuals included in the study, with individuals grouped by serostatus to *Bartonella* sp.. Valid percentages are illustrated.

|  | ***Bartonella* spp. seronegative** | ***Bartonella* spp. seropositive** | **Comparative statistics**  **(p values)** |
| --- | --- | --- | --- |
|  | N=126 | N=21 |  |
| **Median age (IQR) [years]** | 46.0 (34.0-55.0) | 46.0 (40.0-54.0) | 0.62 |
| **Sex** |  |  | 0.25 |
| **Male** | 98 (78.4%) | 19 (90.5%) | . |
| **Female** | 27 (21.6%) | 2 (9.5%) | . |
| **Country of origin** |  |  | 0.07 |
| **Germany** | 53 (44.9%) | 8 (40.0%) | . |
| **Directly neighboring countries** | 31 (26.3%) | 10 (50.0%) | . |
| **Non-directly neighboring countries** | 34 (28.8%) | 2 (10.0%) | . |
| **Time of homelessness [months]** | 12.0 (3.0-48.0) | 54.0 (8.0-132.0) | 0.02 |
| **Sleeping in homeless assistance facilities** | 88 (75.2%) | 15 (78.9%) | 1.00 |
